# Minimizing social bias with sequential collaboration: the role of contributor features in dependent judgments

**DOI:** 10.1038/s41598-025-12227-9

**Published:** 2025-07-24

**Authors:** Maren Mayer, Joachim Kimmerle

**Affiliations:** 1 Leibniz-Institut für Wissensmedien (Knowledge Media Research Center), Schleichstraße 6, 76072 Tübingen, Germany; 2 Department of Psychology, University of Tübingen, Tübingen, Germany

**Keywords:** Human behaviour, Scientific data

## Abstract

Collaborative online projects rely on sequential collaboration; a process in which entries are adjusted consecutively by contributors. Sequential collaboration has recently been examined for numerical judgment aggregation for which it produces highly accurate information. However, the impact of additional information about previous contributors on subsequent judgments remains unclear. Thus, Experiments 1A (N = 85), 1B (N = 186), and 1C (N = 302) examined the effect of presented expertise, gender, and group membership of the previous participant, respectively, on participants’ behavior in sequential collaboration. We did not find a significant effect of any of the presented features on change probability or change magnitude. In Experiments 2 (N = 538) and 3 (N = 878), we focused on previous participants’ expertise as a highly relevant presented feature. We did not find an effect of previous participants’ expertise in Experiment 2. Experiment 3 revealed significant but small effects below the smallest effect size of interest. Overall, these findings suggest that sequential collaboration shows some robustness against simple social influences highlighting its potential as a method for judgment aggregation. This study adds to the understanding of how collaborative processes can be optimized for accuracy and reliability. For collaborative projects these results further emphasize that sequential collaboration is a successful and bias-preventing way of collaboration.

## Introduction

Collaboration has vastly changed with online collaborative projects over the last two decades. Instead of face-to-face communication or collaboration in small groups, these projects are accessible to every interested collaborator at any time around the globe. To organize collaboration, online collaborative projects typically implement a sequential chain. The first contributor creates an entry that is then presented to potential following contributors. Each person encountering this entry decides whether to adjust or maintain the presented entry. If they decide to adjust the entry, it is updated and the updated version is presented to following potential contributors. Previous research has demonstrated that both Wikipedia^1–3^ and OpenStreetMap^4,5^, which use sequential collaboration to organize entries, yield highly accurate information.

Beyond text-based approaches, sequential collaboration may also involve numerical judgments, for example, when contributing the year in which a battle took place or the number of soldiers on each side in Wikipedia, or the coordinates of landmarks in OpenStreetMap. Moreover, sequential collaboration for numerical judgments may also occur in other settings, such as the marketing team in a company collaboratively deciding on the price for a new product or a team estimating the duration of a new software-development project.

Recently, sequential collaboration has been examined in the context of numerical judgments^6–8^, see also^9^. Mayer and Heck^7^ found that over the course of a sequential chain presented judgments are less frequently adjusted, adjustments become smaller, and judgments become more accurate. The final estimates in such a sequential chain are similarly accurate or even more accurate than the average of the same number of independent judgments (wisdom of corwds)^10,11^. Mayer et al.^6^ demonstrated that highly accurate judgments and estimates in sequential collaboration are due to contributors implicitly weighting their judgments by their own expertise. Whereas individuals with lower expertise more often refrain from adjusting presented judgments and improve only moderately inaccurate judgments, individuals with higher expertise more often adjust presented judgments and improve even judgments with little inaccuracies. This effect is especially pronounced in sequential chains where final estimates become more accurate the more and the later experts enter sequential chains. Moreover, Mayer at al.^8^ showed that the advantage of sequential collaboration over aggregated independent judgments is based on both the sequential process of judgment aggregation per se and the possibility to refrain from providing a judgment in the sequential chain.

Even though sequential collaboration shows promising results as a tool for judgment aggregation, contributors in these studies only received minimal information about the previous contributor, namely their judgment. This may not be the case in online collaborative projects where contributors can provide various information about themselves on profile pages. Thus, not only features of the contributors themselves and provided information about the features of the sequential chain influence behavior in sequential collaboration. Information about features of previous contributors may exhibit additional effects on contributors. Previous studies demonstrated that expertise as a feature of the contributors themselves substantially impacts frequency and magnitude of subsequent changes^6^. Moreover, information about the sequential chain provided as information about how often the presented judgment has been maintained previously affected contributors’ behavior in sequential collaboration^12^. However, research is lacking that examines how information about the features of previous contributors influences contributors’ subsequent behavior in a sequential chain.

In the following, we will introduce previous findings on how numerical judgments provided by others are perceived and used differently depending on features such as expertise and social status of these individuals. For sequential collaboration, we similarly assume that information about features of a previous contributor influence subsequent judgments. In five experiments, we investigate an effect of such features on behavior in sequential collaboration over and above already established effects of participants’ expertise and accuracy of the presented judgment. These features comprise expertise, gender, and group membership in a minimal group paradigm, respectively.

## Information about previous contributors

Information about others’ group membership, gender, expertise, or social status, even if this information is unrelated to the task at hand, can influence individuals’ judgments and opinions. Probably the most prominent example of such an influence is described in the elaboration likelihood model that describes under which conditions superficial features can persuade individuals into attitude change^13^.

However, also group membership influences individuals in their evaluation of group members^14^. Known as ingroup bias or ingroup favoritism, individuals tend to favor ingroup members and evaluate ingroup members more positively^14^, which in turn affects cooperation, learning, and advice taking^15–17^. Advice taking is a paradigm closely related to sequential collaboration that also focuses on dependent numerical judgments^18,19^. In a typical advice-taking scenario, individuals are confronted with a numerical judgment question for which they provide an independent judgment. Afterwards, they receive advice from another person, typically another participant, before providing a revised judgment in which they are free to incorporate the advice as much as they like. Individuals in this paradigm typically show egocentric discounting by incorporating advice into their final judgment in a less than optimal way. Van Swol et al.^16^ demonstrated that advice is less taken from members of the outgroup than from members of the ingroup even though this advice is especially valuable. Beyond mere advice taking, group membership also influences individuals in more complex interactions, such as creating Wikis, even when group membership is entirely irrelevant for the content of the wiki text^17^. Here, individuals showed less knowledge integration if the information was provided by outgroup rather than ingroup members.

Beyond group membership, other features can also influence individuals in situations beyond attitude change, such as advice taking. Overall, expert advice is taken more than novice advice^18–20^. Moreover, perceived expertise can be a function of social status and past performance, indicating that these factors are highly interrelated^21^. However, expectations about expert advisors can be exaggerated and only change slowly resulting in too much advice taking from these sources^22^.

Based on these findings, we assumed that information about features of previous participants also shows substantial effects in sequential collaboration. Even though we expected irrelevant features, such as gender or membership in arbitrary groups, to show smaller effects than relevant features, such as expertise or status, we expected that both irrelevant and relevant features of previous participants, if presented in sequential collaboration, influence contributors’ behavior.

To examine these effects, we focus on how much participants align their judgment with the judgment of the previous participant. We assumed that the stronger the influence of the previous contributor, the more participants orient themselves on the judgment of the previous contributor. To measure this influence, we examined how likely contributors are to change the presented judgment of a previous participant and how large their change is if they decide to make an adjustment.

## Experiments 1A, 1B, and 1C

In Experiments 1A, 1B, and 1C, we examined the effect of providing information about features of previous contributors with a minimal manipulation. Information about the previous contributor was presented as an icon together with the judgment of this previous contributor. Such a manipulation is not unrealistic as in collaborative online-projects, information about other users is available but only at the author page and not directly in the editing mode. Change histories typically only contain user names that may provide only little information about features of the previous contributor.

### Experiment 1A

In this experiment, participants were presented with information about the expertise of the alleged previous contributor in sequential collaboration. We expected that participants receiving information that the previous contributor was an expert adjust the presented judgment less often (change probability) and make smaller changes (change magnitude) compared to participants receiving information that the previous contributor was a novice. We expected change probability and change magnitude to range in between if no information about previous participants’ expertise was presented. Since the influence of contributors’ own expertise and the accuracy of the presented judgment have already been established as influences on contributors’ behavior in sequential collaboration^6^, we examine the effects of information about the previous contributor above and beyond these established effects.

Expertise is a domain-specific^23^ and multi-faceted construct^24^. It comprises task-related abilities, skills, knowledge, and experience^25,26^. Experts work on tasks qualitatively differently^26,27^and typically show better performance than novices^28,29^. In this experiment, we manipulated the skills of participants by training them in a technique that allows them to solve the task more easily.

This experiment was preregistered (https://aspredicted.org/jy64-7mv7.pdf). In addition to the preregistration, we also examined the influence of information on previous contributors’ expertise on change magnitude. Materials, data, and analysis scripts for this and all following experiments including an analysis of judgment improvement as additionally preregistered for this experiment can be found in the data repository at OSF (https://osf.io/4q9cz/).

#### Methods

The experiment followed a 2 (participants’ expertise) $\times$ 5 (accuracy of the presented judgment) $\times$ 3 (presented expertise of previous contributor) mixed design. Participants’ expertise was manipulated between participants whereas the accuracy of the presented judgment and the presented expertise of the previous contributor was manipulated within participants.

The material and procedure of the experiment including the manipulation of participants’ expertise and accuracy of the presented judgment were taken from Mayer et al.^6^ Experiments 2 and 3. In this paradigm, participants were asked to provide a judgment for the number of dots (100-599) depicted in a $600 \times 600$ image with white background. A total of 50 pregenerated images were presented, five as exercise, five as manipulation check, and 40 in the sequential-collaboration task of which 30 were analyzed and ten were included for motivational purposes. After consenting to participate in the study, participants were randomly assigned to the manipulation of their expertise. Participants assigned to the expertise condition learned a raster scanning technique. This training aimed at manipulating participants skills to help them provide more accurate judgments. Participants in the novice condition (control condition) read an essay about the importance of accurate numerical judgments. Afterwards, participants answered four control questions to check their attendance to the manipulation. We excluded participants in the expertise condition who answered less than three of these questions correctly from continuing with the study. Next, all participants provided five independent judgments in an exercise, participants in the expertise condition additionally saw a raster on top of the images whereas participants in the control condition did not. To check our manipulation, participants provided independent judgments for another five items, this time with no assistance in both conditions. For all these trials (example and manipulation check), participants had a maximum of 60 s to complete the task to prevent them from simply counting the dots.

Before continuing to the sequential-collaboration phase, participants were instructed that they would now be presented with judgments of previous contributors which they could adjust or maintain. In each trial, they would additionally receive information about the expertise of this previous participant, who was either an expert, a novice, or no expertise information was available. Previous participants’ expertise was presented by an icon indicating whether this participant received a training to provide more accurate judgments or if they received no such training. If no icon was shown, there was also no information regarding the previous participants’ expertise. However, participants did not encounter judgments of previous participants but rather preselected judgments. These preselected judgments deviated +/-70%, +/-35%, or 0% from the correct answer, thus differing in their accuracy. After this instruction, participants completed 30 trials in which they performed sequential collaboration. Thereby, they were presented with the judgment of a previous participant and the additional expertise information. Then, participants decided whether to adjust or maintain the presented judgment and provided a revised one if they decided to do so. Note that in contrast to advice taking participants performing sequential collaboration do not provide initial independent judgments. Instead, they were directly presented with the task and the alleged judgment of a previous participant in each trial. To ensure participants motivation, ten motivational trials for which images only depicted between 10 and 59 dots were presented alongside the test trials. The low number of dots displayed allowed participants to count the dots which showed in a pretest to be less exhausting than estimating. Test trials and motivational trials were presented together in random order. All trials were time restricted such that participants had to remain at least 2 seconds on a page when performing sequential collaboration and a maximum of 60 seconds on every page containing a judgment trial. As sequential collaboration allows to opt out of providing a judgment, the lower time limit served as a restriction for participants to click through the study. The upper time limit was set to ensure participants could not count all dots but had to make an estimate. On average it took participants 21.01 seconds to complete the task ($SD =$ 12.20) After completing the 40 sequential-collaboration trials (30 test trials and ten motivational trials), participants provided demographic information, were debriefed and thanked for participation.

We collected data from 85 student participants who were recruited via the mailing list of the University of Tübingen and participated in exchange for taking part in a gift card lottery. There were no exclusions based on preregistered criteria. In the final sample, 77.65% of participants were female and 100.00% indicated a high school diploma or a university degree as their highest educational degree. Participants had a mean age of 24.66 years ($SD = 6.64$).

#### Results and discussion

Before analyzing the data for our hypotheses, we excluded single trials of participants who did not continue to the next task within 60 seconds. This procedure was preregistered. We excluded 26 trials from our data which resulted in a total of 4,219 trials for the analysis of which 422 were collected in the manipulation-check phase and 2,537 in the sequential-collaboration phase.

**Fig. 1:**
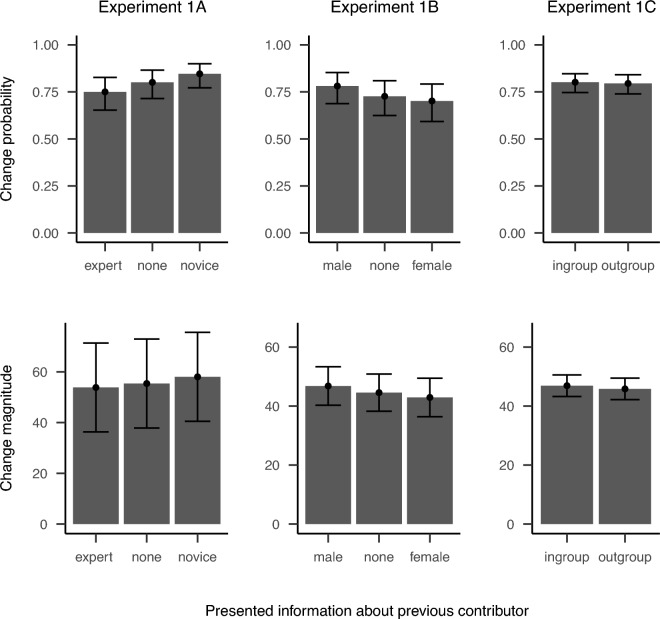
Results for change probability and change magnitude in Experiments 1A, 1B, and 1C. Bars display estimated marginal means and corresponding confidence intervals based on the complex models including the presented feature of the alleged previous participants. Change magnitude is provided as absolute relative change magnitude for Experiment 1A and in pixels for Experiments 1B and 1C.

We used (generalized) linear mixed models with random intercepts for participants and items to test our hypotheses. In line with recent recommendations^30,31^, we added random slopes for the deviation of the presented judgment from the correct answer over and above the random intercepts preregistered. This did not alter the results but ensured that we accounted for variation in the effect of deviation within participants. Even though not explicitly stated in our preregistration, we applied mean-centered contrasts to all independent variables. Thereby, we ensured that the intercept of our models reflect the sample mean across all participants and follow the analysis of Mayer et al.^6^ for the replication. Moreover, we also followed recommendations to test hypotheses as specific as possible^32,33^ which especially applies for the contrast on presented deviation. For participants’ expertise, the condition in which participants were introduced to raster scanning was coded as 0.5, the control condition was coded as -0.5. For deviation of the presented judgment from the correct answer, we set two contrasts to test for a v-shape. This contrast was set as the accuracy of the presented judgment is operationalized to deviate both positively and negatively from the correct answer. Moreover, we implemented a contrast to test for different slopes on both sides of the v-shape. For presented expertise of the previous contributor, we set two contrasts to compare both experts (experts: 0.5, neutral: -0.5) or novices (novices: -0.5, neutral: 0.5) with the neutral condition. The results for the presented information about previous participants’ expertise are depicted in Fig. 1.

First, we conducted a manipulation check to ensure that participants who were trained with raster scanning to become experts indeed provided more accurate judgments than participants in the control condition. To this end, we computed a linear mixed model with absolute relative judgment error of the independent individual judgments provided in the manipulation-check phase in the beginning of the experiment as a dependent variable and expertise condition as an independent variable. Absolute relative error was computed as the absolute difference between provided judgment and correct answer divided by the correct answer and multiplied by 100 to obtain values that represent a percentage deviation from the correct answer. Thus, judgment errors become comparable across trials independent of the number of presented dots in the image. Larger deviations indicate less accurate judgments with an absolute relative error of zero indicating a correct answer. In line with our manipulation, we found a significant negative effect of expertise condition on the accuracy of the provided judgments (${\hat{\beta }} = -18.07$, 95% CI $[-27.28, -8.87]$, $t(83.28) = -3.85$, $p < .001$). Estimated marginal means indicate that participants who received the raster scanning training showed less error ($M = 23.44\%$) than participants in the control condition ($M = 41.51\%$).

To test our hypotheses, we applied a two-step process for each dependent variable. We first computed a simple model only including participants’ expertise, presented deviation and their interaction as independent variables and a complex model adding presented information on the previous participant to the simple model, before comparing those two models. Thereby, we tested whether the presented information about the previous participant has predictive value over and above participants’ expertise and presented deviation. The model showing better model fit was selected to report our results.

To test the hypotheses for change probability, we computed a generalized linear mixed model using a logit link function to capture whether participants made an adjustment to the presented judgment (coded as 1) or maintained the presented judgment (coded as 0). The complex model including effects of presented expertise did not show better fit to the data than the simple model including only participants’ expertise and judgment deviation ($\chi ^2(4) = 4.72, p = .317$). Moreover, the effects of presented expertise in the complex model were not significant (comparison expert-neutral: ${\hat{\beta }} = -0.60$, 95% CI $[-1.28, 0.08]$, $z = -1.72$, $p = .085$, comparison novice-neutral: ${\hat{\beta }} = -0.61$, 95% CI $[-1.31, 0.08]$, $z = -1.73$, $p = .083$, interaction of participants’ expertise and presented expertise expert-neutral: ${\hat{\beta }} = -0.26$, 95% CI $[-0.83, 0.31]$, $z = -0.90$, $p = .370$, interaction of participants’ expertise and presented expertise novice-neutral: ${\hat{\beta }} = -0.03$, 95% CI $[-0.62, 0.55]$, $z = -0.11$, $p = .914$). For the simple model, we found a significant v-shaped effect of deviation (${\hat{\beta }} = 4.86$, 95% CI $[3.52, 6.19]$, $z = 7.13$, $p < .001$), an interaction of expertise and deviation (${\hat{\beta }} = 2.52$, 95% CI $[0.98, 4.05]$, $z = 3.20$, $p = .001$), but no main effect of expertise (${\hat{\beta }} = 0.39$, 95% CI $[-0.05, 0.83]$, $z = 1.75$, $p = .081$). The results for change probability indicate that presented judgments are more frequently adjusted the more they deviate from the correct answer. This slope is even stronger for experts than for novices resulting in less changes to correct presented judgment and more changes to incorrect presented judgments by experts compared to novices.

To test the hypotheses for change magnitude, we computed this dependent variable similar to the absolute relative error for the manipulation check. To obtain change magnitude, we computed the absolute difference between the provided and the presented judgment which was divided by the presented judgment and multiplied by 100. Thereby, we obtain a comparable measure for change magnitude across all trials independent of the number of dots that were presented. If participants did not adjust the presented judgment, change magnitude was zero. Next, we computed the two linear mixed models with change magnitude as dependent variable. The complex model did not show better fit to data than the simple model ($\chi ^2(4) = 3.55, p = .470$) and effects of presented expertise in this model were not significant (comparison expert-neutral: ${\hat{\beta }} = -3.83$, 95% CI $[-30.59, 22.92]$, $t(25.00) = -0.28$, $p = .781$, comparison novice-neutral: ${\hat{\beta }} = -4.55$, 95% CI $[-31.31, 22.21]$, $t(25.00) = -0.33$, $p = .742$, interaction of participants’ expertise and presented expertise expert-neutral: ${\hat{\beta }} = 9.24$, 95% CI $[-1.10, 19.57]$, $t(2337.24) = 1.75$, $p = .080$, interaction of participants’ expertise and presented expertise novice-neutral: ${\hat{\beta }} = 7.22$, 95% CI $[-3.11, 17.54]$, $t(2337.19) = 1.37$, $p = .171$). The simple model revealed significant effects of the v-shaped contrast for deviation (${\hat{\beta }} = 149.83$, 95% CI $[109.10, 190.56]$, $t(31.89) = 7.21$, $p < .001$) as well as a significant interaction of deviation and participants’ expertise (${\hat{\beta }} = 52.19$, 95% CI $[24.09, 80.29]$, $t(89.72) = 3.64$, $p < .001$), but no main effect of participants’ expertise (${\hat{\beta }} = 4.17$, 95% CI $[-3.65, 11.98]$, $t(83.09) = 1.04$, $p = .299$). Similar to change probability, change magnitude increases with larger deviations of the presented from the correct judgment. Moreover, the interaction of expertise and deviation reveals that experts make smaller changes than novices to correct presented judgment and larger changes to incorrect judgments.

Overall, previous findings for this paradigm were mostly replicated as both contributors’ expertise and deviation of presented judgment contributed to change probability and change magnitude in sequential collaboration. However, we did not find a main effect of participants’ expertise on both change probability and change magnitude. This main effect seems to be masked by the interaction such that experts not only make more frequent and larger adjustments to less accurate presented judgments than novices, but also make less frequent and smaller adjustments to highly accurate judgments. Contrary to our expectations, we found no effect of the presented expertise of the previous contributor for both change probability and change magnitude in the study.

### Experiment 1B

In this experiment, we examined the effect of presenting the gender of the previous contributor on the changing behavior in sequential collaboration using a city-location task. Men are typically viewed as more agentic and competent whereas women are typically viewed as more communal and warm^34^. These stereotypes do not only affect the perception of individuals but also shape their behavior and behavior towards them, e.g., in the workplace^35^, in media outlets^36^, or in society^37^. Also for advice taking, there are hints that advice is valued more from male than from female advisors^38^. Even though gender stereotypes seem to decline over the past decades, they are remarkably stable across different groups, such that male and female individuals have highly similar gender stereotypes^39^. Nonetheless, gender stereotypes are still strong in implicit measures^40^. For the task at hand, male participants provided more accurate judgments in past experiments than female participants^7^. Thus, we expected that the gender of previous participants would influence subsequent changing behavior.

Above and beyond effects of participants’ expertise and deviation of the presented judgment from the correct judgment, we thus expected that judgments of men are less often adjusted and adjustments are smaller compared to no information about previous participants’ gender. We expected the opposite pattern arises for presented judgments of women.

In this experiment, we adopted a knowledge-related perspective on expertise. Instead of manipulating participants’ expertise, we measured their task-related knowledge before they performed sequential collaboration.

We again preregistered this experiment (https://aspredicted.org/njcn-5crw.pdf). In addition to the preregistration, we also examined change magnitude as additional dependent variable.

#### Methods

The experiment followed a 3 (previous participant’s presented gender) $\times$ 4 (accuracy of presented judgment) within-participants design. Participants’ expertise was measured in this experiment.

We adapted the city-location task from Mayer et al.^6^ Experiment 1 in which participants provide location judgments for 57 European cities on seven different maps which had $800 \times 500$ pixels. These maps depicted Germany, France, Italy, Austria and Switzerland, Spain and Portugal, the United kingdom and Ireland, and Poland, the Czech Republic, Hungary and Slovakia, respectively. The sea was depicted in blue, countries of interest were depicted in white, and other countries were depicted in grey with black lines indicating borders. After consenting to taking part in the experiment, participants first provided independent individual judgments for 17 cities taken from all seven maps to measure their expertise. After the expertise measurement, we manipulated the presented gender in the following sequential-collaboration phase. To this end, participants were informed that they would be presented with judgments of previous participants in the following. In addition to the judgment, they would also receive information about the gender of the previous participant (either male, female, or no information). This information was made explicit by an either red or blue colored pictogram depicting either a female or male person on the top right of the screen. Additionally, the dot on the map showing the previous judgment was either red or blue. After the manipulation, participants performed sequential collaboration for the remaining 40 items. In each trial they were presented with the judgment of a previous particpant and information about their gender and decided whether to maintain the presented judgment or adjust it and provide a revised judgment. As in the previous experiment, presented judgments were not provided by previous participants but were preselected to deviate 0, 40, 80, or 120 pixels from the correct city location. The presented deviation was randomly assigned for each item and balanced across presented gender. To prevent participants from both clicking through the study and looking up the correct location of the presented cities, all trials had a minimum time of two seconds and an upper time limit of 40 seconds. Participants needed 8.18 seconds on average to provide a judgment for a city location ($SD =$ 6.83). Moreover, we tracked window changes and excluded participants from continuing with the study if they switched the browser window more than five times against explicit instructions. After completing all trials, participants provided demographic information, were debriefed and thanked.

187 students from the University of Tübingen completed the study in exchange for taking part in a gift card lottery. Due to unexpectedly high interest in the study, we terminated data collection after 30 hours before we started data analysis. We excluded 1 participant according to preregistered exclusion criteria since they positioned more than 20% of their judgments outside the highlighted countries of interest. The final sample comprised 186 participants with a mean age of 24.39 years ($SD = 4.53$) of which 52.15% were female and 97.85% indicated a high school diploma or a university degree as their highest educational attainment.

#### Results and discussion

As preregistered, we excluded 279 single trials in which judgments were positioned outside the area of interest and 41 single trials in which judgments were timed out after 40 seconds. This resulted in 10,285 trials for data analysis, 2,902 trials for measuring expertise and 7,383 trials for sequential collaboration.

To test our hypotheses, we again tested for an effect of gender of the alleged previous participant over and above the effect of contributors’ expertise and deviation of the presented judgment from the correct judgment. As in Experiment 1a, we computed (generalized) linear mixed models with random intercepts for participants and items. Additionally, we included a random slope of presented deviation to the models even htough this was not preregistered. We first compared a complex and a simple model before reporting results from the chosen model. Moreover, beyond our preregistration we implemented a mean-centered linear contrast for presented deviation and two mean-centered contrasts comparing either male (male: 0.5, neutral: $-0.5$) or female (female: $-0.5$, neutral: 0.5) alleged previous contributors with the neutral condition. Participants’ expertise was computed as the mean Euclidean distance measured in pixels between the provided judgment and the correct answer of the first 17 independently provided judgments multiplied by $-1$. Thus, larger values indicate higher expertise and lower values indicate lower expertise. Again, results concerning the effect of the presented gender of previous participants are displayed in Fig. 1.

For change probability, the complex model including the effect of gender did not show a better fit to the data than the simple model including only participants’ expertise and judgment deviation ($\chi ^2(4) = 2.41, p = .661$). Moreover, effects of presented gender in the complex model were not significant (comparison female-neutral: ${\hat{\beta }} = -0.48$, 95% CI $[-1.16, 0.21]$, $z = -1.36$, $p = .173$, comparison male-neutral: ${\hat{\beta }} = -0.12$, 95% CI $[-0.79, 0.56]$, $z = -0.34$, $p = .734$, interaction of participants’ expertise and presented expertise female-neutral: ${\hat{\beta }} = -0.06$, 95% CI $[-0.23, 0.11]$, $z = -0.71$, $p = .480$, interaction of participants’ expertise and presented expertise male-neutral: ${\hat{\beta }} = -0.04$, 95% CI $[-0.21, 0.12]$, $z = -0.52$, $p = .603$). The simple model revealed a significant effect of expertise (${\hat{\beta }} = 0.61$, 95% CI $[0.37, 0.84]$, $z = 4.99$, $p < .001$), a significant effect of deviation of presented judgments (${\hat{\beta }} = 1.78$, 95% CI $[1.60, 1.95]$, $z = 20.02$, $p < .001$) and a significant interaction (${\hat{\beta }} = 0.63$, 95% CI $[0.47, 0.78]$, $z = 7.90$, $p < .001$). In line with previous findings, these results show that changes are made more frequently by contributors with higher expertise and for presented judgment with decreasing accuracy and thus more deviation. The interaction effect demonstrates that the increased change probability for increasing presented deviation is even steeper with increasing expertise of participants.

Change magnitude was computed as the Euclidean distance between the presented and the provided judgment. For this dependent variable, the complex model did not show better fit to the data than the simple model ($\chi ^2(4) = 4.03, p = .403$) and effects of presented gender in the complex model were not significant (comparison female-neutral: ${\hat{\beta }} = -4.09$, 95% CI $[-14.45, 6.27]$, $t(36.99) = -0.77$, $p = .444$, comparison male-neutral: ${\hat{\beta }} = -0.42$, 95% CI $[-10.59, 9.75]$, $t(36.96) = -0.08$, $p = .936$, interaction of participants’ expertise and presented expertise female-neutral: ${\hat{\beta }} = -2.30$, 95% CI $[-4.82, 0.21]$, $t(7151.51) = -1.80$, $p = .072$, interaction of participants’ expertise and presented expertise male-neutral: ${\hat{\beta }} = -1.24$, 95% CI $[-3.70, 1.22]$, $t(7150.10) = -0.99$, $p = .322$). The simple model showed a significant main effect of expertise (${\hat{\beta }} = 5.39$, 95% CI $[3.76, 7.02]$, $t(184.05) = 6.47$, $p < .001$), a significant main effect of deviation (${\hat{\beta }} = 49.78$, 95% CI $[47.79, 51.76]$, $t(184.28) = 49.12$, $p < .001$), and a significant interaction of both (${\hat{\beta }} = 13.25$, 95% CI $[11.26, 15.23]$, $t(183.72) = 13.08$, $p < .001$). Thus, larger changes were made with increasing expertise and with increasing deviation of presented judgment from the correct answer. The interaction indicates that the slope for deviation and change magnitude becomes steeper with increasing contributors’ expertise.

These results replicate previous findings for contributors’ expertise and deviation of presented judgment in sequential collaboration. However, we did not find any influence of the presented gender of the previous participant on either change probability or change magnitude.

### Experiment 1C

In this experiment, we examined the effect of group membership induced with a minimal group paradigm on behavior in sequential collaboration using a city-location task. Above and beyond effects of contributors’ expertise and accuracy of the presented judgment, we expected an effect of group membership such that participants make less and smaller changes to presented judgments of members of the ingroup than to presented judgments of members of the outgroup. This experiment was preregistered (https://aspredicted.org/bsc7-vnpt.pdf).

#### Methods

This experiment had a 2 (previous participants’ group membership) x 4 (accuracy of the presented judgment) within-participants design. As we again implemented the city-location task, participants’ expertise was measured.

We used the same city-location task as in Experiment 1B and measured participants’ expertise before they performed sequential collaboration. However, instead of manipulating presented gender, we implemented a minimal group paradigm^41,42^. The minimal group paradigm is a social psychological paradigm that has been developed in the 1960s^42^ and is used to examine ingroup favoritism^43^. Minimal groups in this experiment were created by participants indicating preference for a painting either by Wassily Kandinsky or by Paul Klee^41^ which is one of the earliest manipulations of minimal groups^42^. In the sequential-collaboration phase, participants were presented with the group membership of the alleged previous participants indicated by the respective painting chosen by these participants in the upper left corner of the map.

We sampled data from 309 participants who were recruited via the University’s e-mail service and could participate in a lottery for gift cards in exchange for participation. Due to high interest in this study by participants, we terminated data collection after 12 hours without doing any analyses beforehand. According to preregistered exclusion criteria, we excluded 7 participants who were suspected to have cheated in the study, clicked the same position repeatedly, were timed out in more than 20% of the trials, or positioned more than 20% of their judgments outside the countries of interest. Our final sample comprised 302 student participants with a mean age of 24.59 years ($SD = 6.96$) of whom were 54.64% were female and 99.34% held a high school diploma or a university degree. Overall, 217 participants preferred the painting by Kandinsky whereas 85 preferred the painting by Klee.

#### Results and discussion

In line with the preregistration, we excluded single trials in which judgments were positioned outside the area of interest and were timed out after 40 seconds. Thus, we excluded 446 judgments for being out of areas of interest and 87 judgments for being timed out resulting in 16,669 trials for data analysis (expertise measurement: 4,712 trials, sequential collaboration: 11,957 trials).

We examined the effect of the presented group membership (in this case ingroup or outgroup based on the preferred painting) on change probability and change magnitude over and above participants’ expertise and deviation of presented judgments from the correct answer. We implemented (generalized) linear mixed models for the analysis, and included random intercepts for participants and items. Even though not preregistered, a random slope for presented deviation was added to the model. Beyond the preregistration, we set a mean-centered linear contrast for deviation and mean-centered contrast comparing ingroup ($-0.5$) and outgroup (0.5) were set. Results for the presented group membership of previous participants are displayed in Fig. 1.

For change probability, the complex model including effects of presented group membership did not show better fit to the data than the simple model ($\chi ^2(2) = 0.97, p = .617$), and effects of presented group membership in this model were not significant (comparison ingroup-outgroup: ${\hat{\beta }} = -0.04$, 95% CI $[-0.15, 0.07]$, $z = -0.75$, $p = .455$, interaction of participants’ expertise and group membership: ${\hat{\beta }} = -0.04$, 95% CI $[-0.13, 0.06]$, $z = -0.76$, $p = .446$). Nonetheless, the simple model revealed a significant main effect of expertise (${\hat{\beta }} = 0.71$, 95% CI $[0.53, 0.89]$, $z = 7.70$, $p < .001$), a significant main effect of deviation (${\hat{\beta }} = 1.76$, 95% CI $[1.60, 1.92]$, $z = 21.62$, $p < .001$), and a significant interaction (${\hat{\beta }} = 0.62$, 95% CI $[0.48, 0.75]$, $z = 8.79$, $p < .001$). As found in Experiment 1B, judgments were adjusted more frequently, the more expertise the contributor had, the more the presented judgment deviated from the correct location, and even more the larger both of these factors were.

Examining change magnitude, we did not find a better fit for the complex model than for the simple model ($\chi ^2(2) = 4.56, p = .102$) and effects of presented group membership in the complex model were not significant (comparison ingroup-outgroup: ${\hat{\beta }} = -1.08$, 95% CI $[-2.53, 0.37]$, $t(11552.28) = -1.46$, $p = .145$, interaction of participants’ expertise and group membership: ${\hat{\beta }} = -1.05$, 95% CI $[-2.36, 0.26]$, $t(11599.55) = -1.57$, $p = .117$). However, we found a significant main effect of expertise (${\hat{\beta }} = 7.02$, 95% CI $[5.90, 8.13]$, $t(299.80) = 12.33$, $p < .001$), a significant main effect of deviation (${\hat{\beta }} = 51.28$, 95% CI $[49.55, 53.01]$, $t(299.74) = 58.00$, $p < .001$), and a significant interaction (${\hat{\beta }} = 12.98$, 95% CI $[11.25, 14.71]$, $t(299.10) = 14.70$, $p < .001$) for the simple model. Thus, again, more expertise and larger deviation of the presented judgment from the correct location lead to larger adjustments. This effect is especially pronounced for larger deviations combined with higher expertise.

We were again able to replicate previous findings for sequential collaboration showing positive effects of contributors’ expertise, deviation of presented judgment from the correct answer, and their interaction. However, we did not find evidence for an influence of presented group membership of the previous participant on either change probability or change magnitude.

## Experiment 2

We suspected that we did not find effects of presented features about the previous participant for two reasons. First, the features of the previous participants may not have been presented prominently enough. Providing a short description indicating the possible features of a previous participant may be similar to the presentation of features of previous contributors in online collaborative projects. Both, Wikipedia and OpenStreetMap allow contributors to set up a profile site in which they describe themselves and their expertise in the project. However, in a psychological experiment, participants may have not read this information attentively. This may have also had detrimental impact on the subsequently presented icons that indicated the expertise, gender, or group membership of the alleged previous participant whose judgment was presented. Second, we used a fully crossed design for our within-participants variables. Thus, the accuracy of presented judgments had the same levels for alleged expert, male, and ingroup participants as for alleged novice, female, and outgroup participants. This is unrealistic and may have resulted in that information about the previous participant being discarded altogether.

Therefore, we designed Experiment 2 to address both of these limitations. First, we made the features of the presented previous participants, namely expertise and social status, more prominent in the first place by ensuring participants could only continue the study if they had sufficiently processed them. Moreover, we aligned the presented expertise with the accuracy of presented judgments such that presented judgments were overall more accurate for alleged experts than for alleged novices.

We expected effects of both the expertise and the social status of the alleged previous participant above and beyond participants’ own expertise and the accuracy of the presented judgment. We manipulated previous contributors’ expertise by presenting different occupations. Since occupations naturally vary in the social status that is attributed to individuals in these occupations, we decided to control for social status by explicitly manipulating and analyzing it. Presented judgments provided by previous participants with high expertise or social status should be changed less frequently and changes to these judgments should be smaller than for presented judgments provided by previous participants with low expertise or social status. Moreover, we exploratorily tested if these features of previous participants interacted. This experiment was not preregistered but follows the same methods and analyses as Experiments 1B and 1C.

### Methods

For this experiment, we again used the city-location task with an initial expertise-measurement phase and a subsequent sequential-collaboration phase. However, we manipulated the presented features of the previous participants more explicitly. After the expertise measurement, we presented four vignettes introducing four alleged previous participants. These presented previous participants were all male and middle-aged and only differed in their profession that either came with a high (professor for European geography, truck driver in Europe) or a low (judge for criminal offenses, brick layer) knowledge about European geography and was also associated with either high (professor, judge) or low (truck driver, brick layer) social status. All four vignettes were presented in random order and introduced an icon for each presented person indicating their profession which was then presented in the sequential phase. After introducing the previous participants via vignettes, a manipulation check followed in which we again presented the icons associated with the previous participants and asked participants to match the icons to the presented previous participants. After each answer, the correct vignette was once again repeated. Participants who matched less than three icons correctly could not continue with the study and were excluded at this point. To overcome previous limitations, the deviation of the presented judgment to the correct judgments was aligned with expertise of the presented previous contributor such that presented judgments were overall more accurate when presented with the professor or truck driver rather than with the judge or brick layer. A table detailing this manipulation is available in the Supplemental Material.

We sampled 538 university students for this study. Five participants were excluded due to suspected cheating by looking up answers, clicking repeatedly the same position on the map, positioning more than 20% of judgments out of countries of interest, or being timed out in more than 20% of trials. The final sample consisted of 533 participants with a mean age of 30.79 years ($SD = 13.19$) of whom 53.85% were female.

### Results

In line with Experiments 1B and 1C, we again excluded 120 trials due to timing out after 40 seconds and 446 trials due to positioning judgments outside the area of interest. Thus, 29,439 trials remained for data analysis (expertise measurement: 8,285 trials, sequential collaboration: 21,154 trials).

**Fig. 2:**
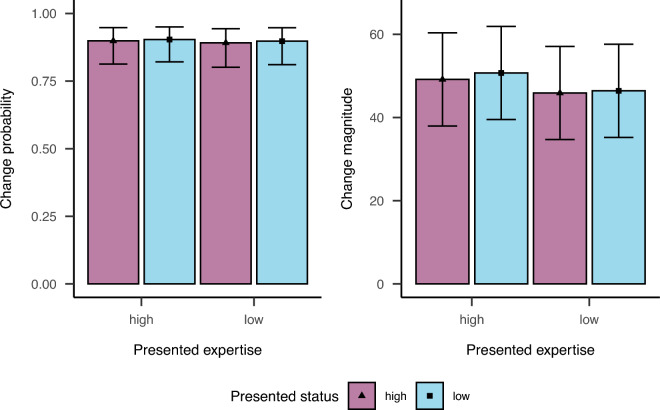
Results for change probability and change magnitude in Experiment 2. Bars display estimated marginal means and corresponding confidence intervals based on the complex models including the presented feature of the alleged previous participants.

To analyze the data for our hypotheses, we used (generalized) linear mixed models with random intercepts for participants and items and also included random slopes for deviation. Moreover, we set a linear mean-centered contrast for presented deviation as well as mean-centered contrasts for presented expertise (high: 0.5, low: $-0.5$) and status (high: 0.5, low: $-0.5$). Finally, we still followed the two-step process detailed above to test for effects of presented expertise and status of the previous contributor over and above contributors’ expertise and presented deviation. Figure 2 displays the results for both change probability and change magnitude.

For change probability, we did not find a benefit of the complex model including effects of presented expertise and status over the simple model including only participants’ expertise and presented deviation ($\chi ^2(3) = 0.07, p = .995$). Additionally, the effects of presented expertise and status in the complex model were not significant (presented expertise: ${\hat{\beta }} = -0.07$, 95% CI $[-0.77, 0.62]$, $z = -0.20$, $p = .839$, presented status: ${\hat{\beta }} = 0.06$, 95% CI $[-0.62, 0.74]$, $z = 0.17$, $p = .864$, interaction of presented expertise and status: ${\hat{\beta }} = 0.01$, 95% CI $[-1.35, 1.37]$, $z = 0.01$, $p = .990$). However, the simple model revealed a significant main effect of contributor’s own expertise (${\hat{\beta }} = 0.45$, 95% CI $[0.25, 0.66]$, $z = 4.32$, $p < .001$), a significant main effect of deviation (${\hat{\beta }} = 2.09$, 95% CI $[1.39, 2.79]$, $z = 5.88$, $p < .001$), and a significant interaction of both (${\hat{\beta }} = 0.53$, 95% CI $[0.42, 0.64]$, $z = 9.41$, $p < .001$). As in the previous experiments, judgment were changed more frequently, the more expertise the contributor had, the more the presented judgment deviated from the correct location, and the larger both of these factors were.

The analysis for change magnitude showed that the complex model including effects of presented expertise and status of the previous contributor did not show better fit to data than the simple model including only participants’ expertise and judgment deviation ($\chi ^2(3) = 0.53, p = .911$). The effects of presented expertise and status in the complex model were also not significant (presented expertise: ${\hat{\beta }} = -3.78$, 95% CI $[-15.10, 7.53]$, $t(35.00) = -0.65$, $p = .517$, presented status: ${\hat{\beta }} = 1.03$, 95% CI $[-10.10, 12.17]$, $t(35.00) = 0.18$, $p = .856$, interaction of presented expertise and status: ${\hat{\beta }} = -1.03$, 95% CI $[-23.29, 21.24]$, $t(35.00) = -0.09$, $p = .929$). Nonetheless, we found a significant main effect of participants’ own expertise (${\hat{\beta }} = 2.66$, 95% CI $[1.81, 3.50]$, $t(530.49) = 6.17$, $p < .001$), a significant main effect of deviation (${\hat{\beta }} = 54.33$, 95% CI $[43.54, 65.12]$, $t(38.46) = 9.87$, $p < .001$), and a significant interaction (${\hat{\beta }} = 11.12$, 95% CI $[9.84, 12.41]$, $t(530.71) = 16.95$, $p < .001$) for the simple model. Thus, higher expertise of contributors and larger deviations of the presented judgment lead to larger adjustments to the presented judgments. This is especially pronounced for larger deviations in combination with higher expertise.

### Discussion

This experiment was designed to examine the influence of expertise and status of the previous contributor that were presented alongside the judgment on the subsequent adjusting behavior in sequential collaboration. We enhanced the manipulation by including vignettes for alleged previous participants who were either high or low on both expertise and status and by aligning the accuracy of presented judgments with the presented expertise of alleged previous participants. With this setup, we were able to replicate previous findings for contributors’ expertise and accuracy of presented judgments. However, we again did not find an effect of the alleged previous participant and therefore of their expertise and social status.

Even though the manipulation of previous participants’ expertise and social status via vignettes was made very explicit (participants had a minimum reading time for the vignettes, they were tested afterwards about them, were excluded if they answered too few questions correctly, and the respective vignette was repeated after each question), the manipulation may still not have been obvious enough. When performing sequential collaboration, participants only saw the presented judgment associated with an icon indicating the previous participant. Since alleged previous participants varied across trials, their influence may have been reduced and the presented judgment itself became more important. Moreover, participants may experience less trust in the information received about the alleged previous participant, if they receive conflicting information. Similar as for algorithms^44^, seeing an alleged expert err, may reduce trust in this information, and participants more strongly rely on the presented judgment and its accuracy. Finally, a student samples may be overall less prone to biases or actively try to counteract them. Thus, university students may not show much bias in line with expertise and social status of alleged previous participants.

## Experiment 3

To address the limitations of Experiment 2, we used a sample from the general public for Experiment 3. Moreover, we again provided a vignette about the alleged previous participant, but made this a between rather than within-participants manipulation. This design was less realistic for online collaborative projects, but was aimed to be helpful to elicit an effect or demonstrate that effects of information about the previous participant are negligible in sequential collaboration. To this end, we performed a power analysis with a smallest effect size of interest (SESOI) to ensure a sufficient sample and conducted a sensitivity analysis for the collected data. This study including the power analysis was preregistered at https://aspredicted.org/bync-7w2t.pdf.

### Methods

For this experiment, we again implemented the city-location task. After the initial expertise measurement, participants were introduced to one of two alleged previous participants; both university students, one studying geography who scored highly in the first 17 items that participants also just completed, one studying mathematics who scored average in these items. Thus, we manipulated the expertise of the alleged previous participant both with their previous performance and their educational experience concerning the task. After participants read the vignette for at least 30 seconds, they completed four manipulation-check questions asking about the vignette. These questions comprised the subject the presented previous participant studied, how they performed in the initial 17 items, which university they were attending, and why participants were presented with this information. It was only possible to proceed with the study if they answered at least three of them correctly. The vignettes and control questions can be found in Appendix B. For the following 40 city-location items, all participants were presented with real judgments of a participant who provided those in Experiment 3 of Mayer, and Heck^7^ and reached percent rank of five. Thereby, we controlled for the influence of accuracy of the presented judgment on the change probability and change magnitude and prevented participants discarding the information about the previous participant due to surprisingly inaccurate presented judgment.

Since we have not found any effects of presenting features of the previous participant in the previous experiments, we conducted a power analysis before data collection. We performed a power analysis for change magnitude as a dependent variable since change magnitude best reflects differences in changing behavior and has more impact on the resulting judgments than change probability. We used *simr*^45^ to conduct a simulation-based power analysis for the model comparison of the simple and complex model as practiced for the analysis of our hypotheses in the previous four studies. The simulation was based on data from Mayer et al.^6^ Experiment 1 which has the same design and also collected data from the general public. We set the SESOI to 5 pixels which is the size of the dot positioned on the map to provide a judgment. The simulation revealed that for this SESOI and a power of $1-\beta = .90$ a sample size of at least 800 participants was required. We aimed at collecting 880 participants to ensure enough participants even after exclusions based on preregistered exclusion criteria. With the 800 participants determined for an effect in change magnitude, we could detect an odds ratio of $2.23$ with a power of $1-\beta = .90$ and an odds ratio of $2.01$ with a power of $1-\beta = .80$ for change probability.

Based on our power analysis, we collected complete data from 889 participants via the German commercial panel provider bilendi. 11 participants were excluded due to suspected cheating or judgments outside countries of interest as preregistered. Thus, the final sample contained 878 participants, of which 502 were introduced to the expert previous participant and 376 were introduced to the average performing previous participant. The imbalance in conditions was due to participants having somehow more difficulties with control questions for the average performing previous participant. Overall, 47.38% of participants were female 52.51%, were male. In contrast to the first four experiments, we collected a more diverse sample in which 51.25% participants had a college degree, 28.70% participants had a high school diploma, 11.96% had vocational education, and 7.97% participants a lower educational attainment. The mean age of our sample was 47.04 years ($SD = 15.13$).

### Results

As preregistered, we excluded 179 trials due to being timed out after 40 seconds. 34,941 trials remained for the analysis of our hypotheses (expertise measurement: 14,724, sequential collaboration: 34,942)

**Fig. 3:**
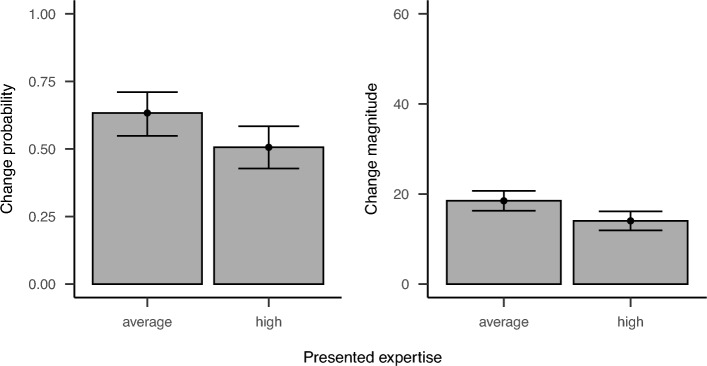
Results for change probability and change magnitude in Experiment 3. Bars display estimated marginal means and corresponding confidence intervals based on the complex models including the presented feature of the alleged previous participants.

We again used (generalized) linear mixed models to test our hypotheses. This time, however, we excluded the random slope for deviation from the models as they did not converge sufficiently. Whereas the accuracy of presented judgments was entered as a continuous predictor, we used a mean-centered contrast for the expertise of the previous participant (high: -0.5, average: 0.5) which was not preregistered. For change probability, the complex model showed better fit to the data than the simple model ($\chi ^2(1) = 5.96, p = .015$). Using the complex model to analyze our data, we did not find a significant main effect of participants’ own expertise (${\hat{\beta }} = -0.32$, 95% CI $[-0.53, -0.10]$, $z = -2.87$, $p = .004$), but as in previous studies a significant positive main effect of deviation (${\hat{\beta }} = 0.01$, 95% CI $[0.00, 0.02]$, $z = 2.53$, $p = .011$), and a significant positive interaction (${\hat{\beta }} = 0.01$, 95% CI $[0.01, 0.01]$, $z = 10.89$, $p < .001$). Additionally, the model revealed a significant positive effect of presented expertise on change probability (${\hat{\beta }} = 0.52$, 95% CI $[0.11, 0.93]$, $z = 2.48$, $p = .013$). As depicted in Figure 3, participants presented with judgments of an alleged expert changed those in 50.62% of trials whereas participants presented with judgments of an alleged novice change those in 63.32% of trials.

For change magnitude, the complex model again showed better fit to the data than the simple model ($\chi ^2(1) = 22.37, p < .001$). Contrary to previous studies and our hypotheses, we found a significant negative effect of participants’ expertise on change magnitude (${\hat{\beta }} = -6.19$, 95% CI $[-7.17, -5.21]$, $t(874.39) = -12.42$, $p < .001$). Nonetheless, as found in previous studies, the model revealed a significant positive effect of deviation (${\hat{\beta }} = 0.23$, 95% CI $[0.14, 0.32]$, $t(40.10) = 5.07$, $p < .001$) and positive interaction of contributors’ expertise and deviation on change magnitude (${\hat{\beta }} = 0.01$, 95% CI $[0.01, 0.01]$, $z = 10.89$, $p < .001$). Additionally, we found a significant positive effect of presented expertise (${\hat{\beta }} = 4.46$, 95% CI $[2.62, 6.29]$, $t(874.36) = 4.76$, $p < .001$) indicating larger changes were made to judgments of an alleged average rather than high performing previous participant (Figure 3). Even though significant, this effect of expertise of the presented previous participant was below the smallest effect size of interest we defined for the power analysis.

### Discussion

In this experiment, we found significant effects of presented expertise on both change probability and change magnitude over and above effects of contributors’ own expertise and deviation of the presented judgment from the correct city location. In line with our hypotheses, participants were more likely to adjust a presented judgment and provided larger adjustments if the presented judgment was from an alleged novice rather than an alleged expert. However, the effect of change magnitude was below the SESOI and thus smaller than the dot that was positioned on the maps by participants. Even though participants seem to act according to presented expertise when deciding to adjust or maintain the presented judgment, this had only little influence on the overall magnitude of changes. This is especially remarkable when considering the extent of our manipulation.

Moreover, we also analyzed the effects of contributors’ own expertise and presented deviation on change probability and change magnitude. Whereas results for presented deviation were in line with our hypotheses, results for contributors’ expertise were not. Contrary to our hypothesis and previous findings, we found no effect of contributors’ expertise on change probability and a negative effect on change magnitude.

These effects are most likely due to the already high accuracy of presented judgments. Participants with higher expertise made smaller changes than participants with lower expertise, which indicates that participants with higher expertise correctly identify the presented judgments as already highly accurate. Thus, they only made small changes to these presented judgments, whereas participants with lower expertise did not seem to be able to recognize the presented judgments as highly accurate. Such a behavior has already been proposed by Mayer et al.^6^ as they hypothesized that experts are not only able to detect and improve inaccurate judgments but also detect already highly accurate judgments and refrain from (large) adjustments in such cases. Even though not in line with our hypotheses, these results concur with expectations for sequential collaboration if presented judgments are highly accurate as in the present study.

## General discussion

In five studies, we examined the effect of features of the previous participants presented in sequential collaboration on the change probability and the change magnitude of entries. For the first four experiments, we were able to replicate previous effects of participants’ expertise and presented judgment’s deviation on both change probability and change magnitude but did not find an effect of any of the presented features of a previous participant. For the last study, we implemented a power analysis with a SESOI to detect even a small effect. This experiment showed an effect of presented expertise of the previous participant for both change probability and change magnitude. However, the effect was rather small for change magnitude and below the smallest effect size of interest.

In these experiments, the presented features of previous participants exhibited only minor influence on participants in sequential collaboration. Only if the manipulation of presented features of previous contributors was very salient and the feature was highly relevant for the task, we were able to find a small effect. However, as already found in previous studies, participants were consistently much more influenced by their own expertise and the accuracy of the presented judgment.

Even though participants performing advice taking are substantially influenced by the adviser’s expertise^18,19^, this does not seem to be the case for sequential collaboration. This may be due to two differences in the paradigm. First, contributors in sequential collaboration do not form an initial independent judgment which has been found to be associated with an increased rate of advice taking^46^. Second, contributors can opt out of providing a judgment and maintain the presented judgment, which has been demonstrated to foster an implicit weighting of judgments by expertise^6^. Even though it is theoretically possible in advice taking to provide the advice as a final judgment and thereby opt out of providing a new judgment, it is unlikely that participants experience this action similar as opting out. Thus, sequential collaboration seems to be designed in such a way that criteria that contribute to how much participants can potentially improve a presented judgment are those that guide participants’ adjustments primarily. Irrelevant criteria, on the other hand, appear to be far less likely to guide decisions of opting out and if they do their influence appears to be limited.

### Limitations and future research directions

Even though our experiments provide important insights and show robust results, this research has some limitations. First, our studies primarily focused on previous participants’ expertise as the feature presented to contributors in sequential collaboration. We did not include all possible features that collaborators may encounter in profiles available in online collaborative projects. Changing behavior in sequential collaboration may be affected by bias blind spots^47^ or elicit motivated reasoning^48^. If contributors expect biases in previous contributors, they are likely to adjust for these. Moreover, contributors may be more likely to revise judgments of others that have opposing opinions on critical topics. However, it remains unclear whether the attribute of a previous contributor elicits bias blind spots or motivational reasoning or the entries themselves. Even though there are findings suggesting that these may root in competence expectations^49^, future research should examine whether and how these biases may operate in sequential collaboration.

Second, we examined the effect of presenting features of the previous participant directly with their judgment. In online collaborative projects, however, such information is not directly available with the presented entry. In contrast, information about previous contributors is stored in separate web pages and only if these contributors decided to set up a page for their user profile. Thus, we expect that the effect of features of a previous contributor are even smaller for online collaborative projects.

Last, we used predominantly map material in the experiments described above. This limits the generalizability of our results. Sequential collaboration has already been successfully examined with various material including a random-dot estimation task^Exp. 1A,^^6^ and general knowledge questions^7^. We therefore expect that the results of our experiment may generalize to other settings and material for sequential collaboration with numerical judgments. Nonetheless, future research should systematically examine the generalizability of our results ideally with high powered studies and smallest effect sizes of interest.

## Conclusion

In five studies we experimentally demonstrated that sequential collaboration is only minimally influenced by additional information about features of previous contributors, such as their expertise, gender, or group membership. Rather contributors’ own expertise and the accuracy of the presented judgment are the primary factors guiding adjustments. These findings contribute to the understanding of judgment aggregation, showing that sequential collaboration has the potential to effectively limit social influences and maintain high accuracy.

## Supplementary Information

Supplementary Information.

## Data Availability

All collected data for the experiments as well as respective R scripts for the analyses are available at the Open Science Framework (https://osf.io/4q9cz/). The present version of the manuscript (2025/07/20 11:51:45) was uploaded to PsyArXiv and ResearchGate for timely dissemination and has not yet been peer reviewed.
