# Supplementary material for: Minimizing social bias with sequential collaboration: the role of contributor features in dependent judgments
Source: Sci Rep. 2025 Jul 24;15:26996. doi: 10.1038/s41598-025-12227-9 (PMC12289943; doi:10.1038/s41598-025-12227-9)
Supplement: Supplementary file 1 — Supplementary Information. [file 41598_2025_12227_MOESM1_ESM.pdf]

Appendix A

Manipulation of expertise in Experiment 2

Table A1

Manipulation of presented expertise for Experiment 2 using map material.

Presented deviation	Professor	Truck driver	Judge	Brick layer
0 pixels	3	3	2	2
40 pixels	3	3	2	2
80 pixels	2	2	3	3
120 pixels	2	2	3	3

Appendix B

Vignettes and control questions for Experiment 3

The following vignettes and were provided to participants in the respective conditions in Experiment 3. All instructions have been translated from German.

High expertise vignette

Please read the following information carefully!

Now begins the second part of the study. Your task in this part of the study is also to mark cities as accurately as possible on maps. On the map, you will see a red dot indicating an entry made by a previous participant in the study.

The participant whose entries you see has also answered the questions you just answered. He was surveyed in an earlier study at the University of Tübingen. He indicated that he is studying geography. Regarding the tasks you just completed, this participant belongs to the top 5% of participants in the earlier survey. This means that 95% of the other participants performed worse. The performance of the previous participant is therefore very high. In each round, you can correct the marked position by clicking on the map. If you do not want to improve the entry, click directly on “Next”.

The “Next” button will appear on this page after 30 seconds.

Low expertise vignette

Please read the following information carefully!

Now begins the second part of the study. Your task in this part of the study is also to mark cities as accurately as possible on maps. On the map, you will see a red dot indicating an entry made by a previous participant in the study.

The participant whose entries you see has also answered the questions you just answered. He was surveyed in an earlier study at the University of Tübingen. He indicated that he is studying mathematics. Regarding the tasks you just completed, this participant belongs to the top 45% of participants in the earlier survey. This means that 55% of the other participants performed worse. The performance of the previous

participant is therefore average. In each round, you can correct the marked position by clicking on the map. If you do not want to improve the entry, click directly on “Next”.

The “Next” button will appear on this page after 30 seconds.

Control questions

What subject is the presented participant studying? a) Geography b) Mathematics c) Business d) Law

How well did the presented participant perform in the first part of the study? a) He was among the best participants. b) He performed average. c) He was among the worst participants.

At which university was the survey conducted in which the described participant took part? a) University of Tübingen b) University of Konstanz c) Technical University Munich d) University of Frankfurt

Why was the participant presented to you? a) I will be presented with his judgments in the following part of the study. b) The participant was the best participant in the study so far. c) I am supposed to write about this participant in the following part of the study. d) This participant has developed a geography test.
